# Phylogeography and demographic history of *Gyrodactylus konovalovi* (Monogenoidea: Gyrodactylidae), an ectoparasite on the East Asia Amur minnow (Cyprinidae) in Central China

**DOI:** 10.1002/ece3.6000

**Published:** 2020-01-18

**Authors:** Tao Chen, Juan Chen, Ling Tang, Xiaoning Chen, Jun Yan, Ping You

**Affiliations:** ^1^ College of Life Sciences Shaanxi Normal University Xi’an China; ^2^ College of Chemistry and Bioengineering Guilin University of Technology Guilin China

**Keywords:** demographic history, *Gyrodactylus konovalovi*, phylogeography, Qinling Mountains

## Abstract

*Gyrodactylus konovalovi* is an ectoparasite on the Amur minnow (*Rhynchocypris lagowskii*) that is widely distributed in the cold fresh waters of East Asia. In the present study, the phylogeography and demographic history of *G. konovalovi* and the distribution of its host in the Qinling Mountains are examined. A total of 79 individual parasites was sequenced for a 528 bp region of the mitochondrial NADH dehydrogenase subunit 5 (ND5) gene, and 25 haplotypes were obtained. The substitution rate (dN/dS) was 0.068 and indicated purifying selection. Haplotype diversity (*h*) and nucleotide diversity (*π*) varied widely in the Qinling Mountains. Phylogenetic trees based on Bayesian inference (BI), maximum likelihood (ML), and maximum parsimony (MP) methods and network analysis revealed that all haplotypes were consistently well‐supported in three different lineages, indicating a significant geographic distribution pattern. There was a significant positive correlation between genetic differentiation (*F*_st_) and geographic distance. The results of mismatch distribution, neutrality test and Bayesian skyline plot analyses showed that whole populations underwent population contraction during the Pleistocene. Based on the molecular clock calibration, the most common ancestor was estimated to have emerged in the middle Pleistocene. Our study suggests for the first time that a clearly phylogeography of *G. konovalovi* was shaped by geological events and climate fluctuations, such as orogenesis, drainage capture changes, and vicariance, during the Pleistocene in the Qinling Mountains.

## Graphical abstract

*Gyrodactylus konovalovi* is an ectoparasite on East Asia minnow *Rhynchocypris lagowskii* that is widely distributed in cold freshwater. In the study, the phylogeographic pattern and demographic history of *G. konovalovi* along with the distribution of its host in the Qinling Mountains have been examined.

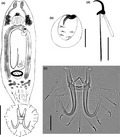

## 1 INTRODUCTION

Phylogeography is the study of historical processes that may be responsible for the contemporary geographic distributions of genealogical lineages within and among closely related species and is primarily conducted using molecular markers (Avise, 2000). Phylogeography can be used to identify the different historical forces, such as population expansion, population bottleneck, climate oscillation, vicariance, and migration, that shape current patterns, analyze the variation in population distributions and reconstruct the evolutionary processes of fauna (Huang, 2012). Recently, animal mitochondrial DNA (mtDNA) permits an extension of phylogenetics referring to the microevolution between systematics and population genetics (Avise et al., 1987). mtDNA is employed due to its maternal inheritance to trace maternal lineage far back in time, rapid mutation rate to track the ancestry of many species back hundreds of generations and the genetic relationships of individuals within species to be determined and low level of intermolecular genetic recombination (Brown, George, & Wilson, 1979; Clayton, 1982; Giles, Blanc, Cann, & Wallace, 1980). mtDNA has been widely regarded as an effective molecular marker in studies of population genetics, phylogeography and comparative phylogeography (Bowen et al., 2016; Hansen, Bakke, & Bachmann, 2007; Hardouin et al., 2018; Huang et al., 2017; Huyse, Oeyen, Larmuseau, & Volckaert, 2017; Li, Shi, Brown, & Yang, 2011; Lumme, Mäkinen, Ermolenko, Gregg, & Ziętara, 2016; Pettersen, Mo, Hansen, & Vøllestad, 2015; Schneider, Cunningham, & Moritz, 1998; Wang, Jiang, Xie, & Li, 2012; Wu et al., 2009; Yu, Chen, Tang, Li, & Liu, 2014). Such studies have revealed the roles of population geographic distribution, population expansion, dispersal, gene flow, climate oscillation, and vicariance in shaping current phylogeographic patterns.

The Gyrodactylidea includes a group of oviparous species from loricarid catfish in South America, and a group of viviparous species that found in bony fish, amphibians, crustaceans, and mollusks (Boeger, Kritsky, & Pie, 2003; Llewellyn, 1984; Paetow, Cone, Huyse, Mclaughlin, & Marcogliese, 2009). More than 400 valid species have been described (Harris, Shinn, Cable, & Bakke, 2004), and representatives are found worldwide, parasitizing the skin, fins and gills of marine, and freshwater fishes, both in the wild and in captivity (Bakke, Cable, & Harris, 2007; Boeger et al., 2003). Recently, three different genera (*Gyrodactylus* von Nordmann, 1832; *Paragyrodactylus* Gvosdev et Martechov, 1953; *Laminiscus* Palsson et Beverley‐Burton, 1983) and 60 species of Gyrodactylidae Beneden et Hesse, 1864 were identified from freshwater fishes in China, including Cypriniformes, Gadiformes, Gasterosteiformes, Mugiliformes, Perciformes, Salmoniformes, and Siluriformes (Chen, 2018; Wu, Lang, & Wang, 2000). Gyrodactylids are highly pathogenic to cultured and wild fishes worldwide (Anttila, Romakkaniemi, Kuusela, & Koski, 2008; Bakke et al., 2007; Razzolini, Murari, Baldisserotto, & Boeger, 2019; Tu, Ling, Huang, & Wang, 2015; You, Easy, & Cone, 2008; You, King, Ye, & Cone, 2014). The lesions caused by parasite adhesion and feeding activity increased host susceptibility to infections by bacteria, fungi and other parasites (Collins, Mo, Buchmann, & Cunningham, 2002; Martins, Shoemaker, Xu, & Klesius, 2011). Large infestations of *Gyrodactylus salaris* Malmberg, 1957 caused extensive damage to the epidermis of Atlantic salmon (*Salmo salar* L.), which led to secondary infections and, ultimately, mortality (Jansen, Matthews, & Toft, 2007; Pettersen, Hytterød, Vøllestad, & Mo, 2013). The presence of hundreds of specimens of *Gyrodactylus kobayashii* Hukuda, 1940 was the primary reason for around most mortality in the goldfish (*Carassius auratus* L.) from a fishpond in central China (Tu et al., 2015). Appropriate measures prevent the spread of the parasite and epizootics in rivers and farms, primarily via chemical and physical disinfection (Eriksen & Pettersen, 2016; Koski, Anttila, & Kuusela, 2016; Steverding, Morgan, Tkaczynski, Walder, & Tinsley, 2005; Tu, Huang, Hu, Ling, & Wang, 2018; Tu, Ling, Huang, Zhang, & Wang, 2013).

**Figure 1:**
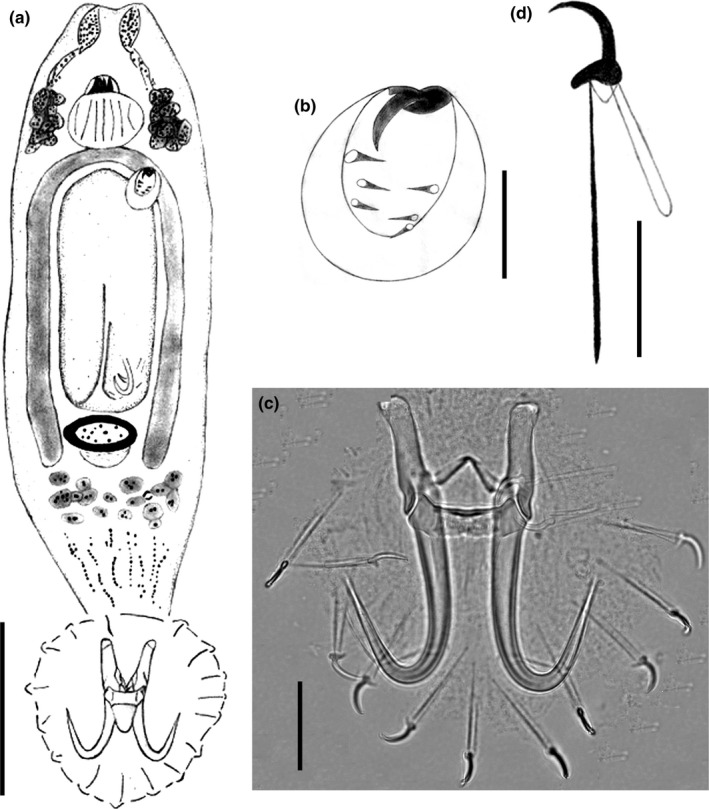
*Gyrodactylus konovalovi* Ergens, 1976 (a) Whole mount specimen. (b) Male copulatory organ. (c) Haptoral complex (accessory hard part, hamuli, ventral and dorsal bar). (d) Marginal hook. Scale bar: a = 100 μm; b = 5 μm; c = 20 μm; d=10 μm

*Gyrodactylus konovalovi* Ergens, 1976 (Figure 1) was described on the fins, skin, and gills of the Amur minnow *Rhynchocypris lagowskii* Dybowski, 1869 in Mongolia and Russia based on the morphological and metrical analyses of the hard parts of the opisthaptor (Ergens, 1976). Although the host *R. lagowskii* and related fishes species were selected for phylogeographic studies due their widespread distributions in cold freshwater from the Lena and the Amur Rivers southward to the Yangtze drainages in East Asia (Hassan, Ishikawa, Seki, & Mahmuda, 2015; Higuchi & Watanabe, 2005; Min & Yang, 1986; Sakai, Ueda, Yokoyama, Safronov, & Goto, 2014; Xue et al., 2017; Yu et al., 2014), there has yet to be a corresponding study of *G. konovalovi* parasitzing *R. lagowskii* in the waters of the Qinling Mountains in central China. The Qinling Mountains have played an important roles in influencing the phylogeography of a variety of organisms including *Feirana quadranus*, *Feirana taihangnica*, *Batrachuperus tibetanus*, *Rosa sericea*, *Pseudorasbora parva*, *R. oxycephalus,* and *Brachymystax lenok tsinlingensis* (Bowen et al., 2016; Liu et al., 2015; Lumme et al., 2016; Wang et al., 2012; Wang, Jiang, Xie, & Li, 2013; Yu et al., 2014)*.* These mountains represent a natural boundary between the northern and the southern regions of the country and separate the Chinese temperate and subtropical climatic zones (Ding, Wang, Zheng, Wang, & Yang, 2013), resulting in differentiated terrestrial and freshwater fauna (Li, 1981; Zhang, 2011). The split dates between species are estimated to be in the middle Pleistocene and may have been a consequence of the rapid uplift (more than 500–1,500 m) of these mountains, which was influenced by the Qinghai‐Tibet Plateau movement (Zhang & Fang, 2012). The Eurasian minnow (*Phoxinus phoxinus* L.) hosts the most diverse fauna of *Gyrodactylus* described on any fish species in the Palearctic region, including 17 species, whereas the Amur minnow *R. lagowskii* hosts three species of *Gyrodactylus* worldwide (Harris et al., 2004). Therefore, these species might be a suitable assembly for studying the ecology and phylogeographic evolution of a host and parasite system without the complications caused by host switching (Ziętara & Lumme, 2002). Recently, there were some phylogeographic studies of the diverse fauna of the genus *Gyrodactylus* in Europe and Brazil, such as *G. thymalli*, *G. arcuatus*, *G. gondae* and *G. corydori*, with obvious genetic structures (Bueno‐Silva, Boeger, & Pie, 2011; Huyse et al., 2017; Lumme et al., 2016; Pettersen et al., 2015).

In this study, we first examined the phylogeography and demographic history of the *G. konovalovi* based on the mitochondrial NADH dehydrogenase subunit 5 (ND5) gene from 79 individuals from eleven populations and its host distribution in the Qinling Mountains in central China, assessed the population genetic differentiation and demographic history and tested how the geological events or climate oscillation during the Pleistocene may have affected the current phylogeographic pattern of this parasite.

## 2 MATERIALS AND METHODS

### 2.1 Ethics statement

This study was approved by the Animal Care and Use Committee of Shaanxi Normal University. The host is not evaluated in IUCN red list status (https://www.iucnredlist.org). None of the species (fish or parasite) sampled is endangered or protected in China (Yue & Chen, 1998). Host sampling was permitted by the local level authority in scientific research.

### 2.2 Sample collection

**Figure 2:**
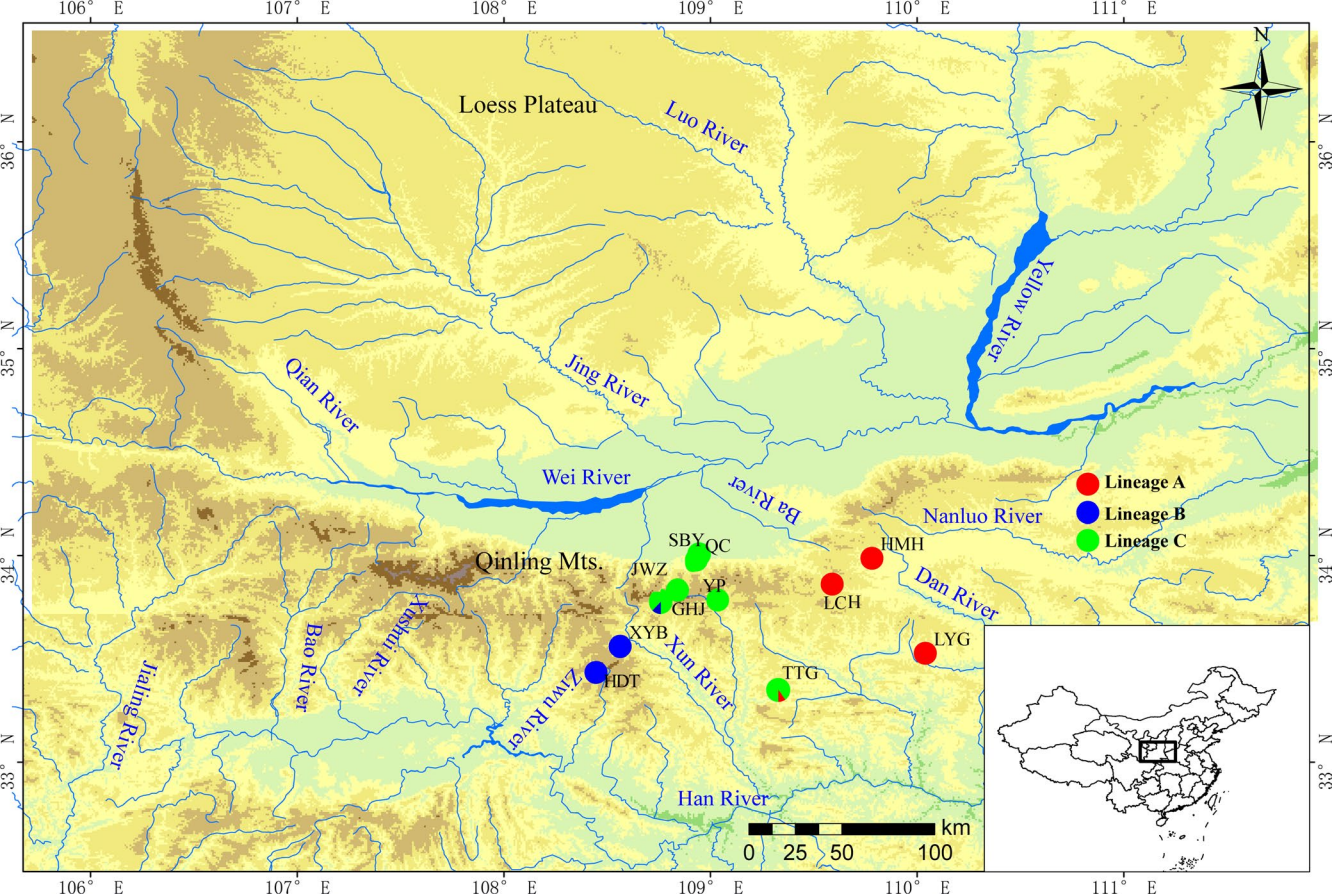
Map of sampling localities for *Gyrodactylus konovalovi* populations. The locality codes are given in Table 1. The lineages labeled red, blue, and green represent for lineages a, b, and c, respectively

Specimens of *R. lagowskii* were sampled from 48 localities, which covered the most Qinling Mountains from May to October in 2016 and 2017. Fish were rapidly euthanized by a blow to the head and stored in 96% ethanol. *Gyrodactylus* species were collected from the skin and fins of unique host under a stereomicroscope in the laboratory, and one parasite specimen per host individual was used to avoid pseudoreplication. Subsequently, the *Gyrodactylus* specimens were examined microscopically, and species identification was performed based on the morphology of the opisthaptor (Ergens, 1976). A total of 79 individuals of *G. konovalovi* from eleven localities were identified (Figure 2 and Table 1). Finally, each parasite specimen and host specimen were individually stored in 96% ethanol at 4°C. Voucher specimens of the parasites and hosts were deposited in the Fish Disease Laboratory, College of Life Sciences, Shaanxi Normal University, Xi'an, China, 710062.

**Table 1:** Sampling information and haplotype diversity based on the ND5 gene for eleven populations of *Gyrodactylus konovalovi*

Population code	Locality	Lineage	*n*	Coordinates	Altitude	Haplotypes	*h*	π
JWZ	Chang'an Co.	C	8	N33.864368°/E108.824842°	1685.1m	Hap2(2), Hap3(1), Hap4(5)	0.607 ± 0.164	0.01333 ± 0.00327
QC	Chang'an Co.	C	5	N33.977764°/E108.932698°	794.6m	Hap12(3), Hap13(1), Hap14(1)	0.700 ± 0.218	0.00568 ± 0.00172
SBY	Chang'an Co.	C	9	N34.005723°/E108.946291°	625.4m	Hap12(1), Hap15(4), Hap16(4)	0.667 ± 0.105	0.00337 ± 0.00095
GHJ	Ningshan Co.	C/B	8	N33.773644°/E108.769971°	1,412.0m	Hap1(1), Hap2(3), Hap3(2), Hap4(1), Hap5(1)	0.857 ± 0.108	0.01907 ± 0.00615
YP	Zhashui Co.	C	10	N33.784181°/E109.035646°	1,106.2m	Hap4(1), Hap13(1), Hap21(1), Hap22(1), Hap23(1), Hap24(1), Hap25(4)	0.867 ± 0.107	0.00585 ± 0.00152
TTG	Zhen'an Co.	C/A	10	N33.363943°/E109.304154°	1,053.1m	Hap7(1), Hap17(2), Hap18(6), Hap19(1)	0.644 ± 0.152	0.02361 ± 0.01731
XYB	Ningshan Co.	B	9	N33.559413°/E108.563282°	1,384.3m	Hap1(2), Hap20(7)	0.389 ± 0.164	0.00074 ± 0.00031
HDT	Ningshan Co.	B	7	N33.434294°/E108.445810°	1529.1m	Hap6(7)	–	–
LCH	Shangzhou Co.	A	1	N33.861331°/E109.589170°	1,074.1m	Hap7(1)	–	–
HMH	Shangzhou Co.	A	7	N33.986237°/E109.781073°	843.4m	Hap7(2), Hap8(3), Hap9(1), Hap10(1)	0.810 ± 0.130	0.00433 ± 0.00087
LYG	Shanyang Co.	A	5	N33.526551°/E110.039283°	830.7m	Hap11(5)	–	–

Abbreviations: *n*, the number of individuals; *h*, haplotype diversity; π, nucleotide diversity.

### 2.3 DNA extraction, PCR amplification, and direct sequencing

Total genomic DNA of *G. konovalovi* was extracted from a single individual using the TIANamp Micro DNA kit (Tiangen Biotech, Beijing, China) following the manufacturer's protocol. The ND5 gene sequences of the 79 individuals of *G. konovalovi* from the eleven populations were amplified by polymerase chain reaction (PCR) amplification using a newly specific primers designed based on the congeneric species *Gyrodactylus brachymystacis* and *Gyrodactylus parvae* (Ye, Easy, King, Cone, & You, 2017), the forward primer gkND5‐*F* (5'‐ATTAGAAAGAGAGCAGTGT‐3') and the reverse primer gkND5‐R (5'‐ATTTGTAGATGATAGCAAG‐3') were used to amplify a segment of 528 bp. Each PCR amplification was performed in a total volume of 25 µl, containing 3.0 mM MgCl_2_, 10 mM Tris‐HCl (pH 8.3), 50 mM KCl, 0.25 mM of each dNTP, 1.25 U rTaq polymerase (TaKaRa, Dalian, China), 0.4 μM of each primer, 45 ng gDNA, tapped with Milli‐Q water. The following cycling conditions were applied: initial denaturation for 1 min at 93°C followed by 35 cycles of denaturation for 10 s at 92°C, annealing for 1.5 min at 50°C and extension for 2 min at 60°C with a final extension for 6 min at 72°C. All PCR fragments were initially purified with a PCR purification kit (BGI Biotech, Shenzhen, China), subsequently subjected to electrophoresis in a 1% agarose gel and finally sequenced with PCR forward primer with an ABI Prism^®^ 3,730 automated sequencer (Applied Biosystems, Foster City, USA).

### 2.4 Data analyses

#### 2.4.1 Population genetic diversity

A total of 79 ND5 gene sequences were visually inspected and manually edited using BioEdit v7.0.9.0 (Hall, 1999) and then aligned with MUSCLE (Edgar, 2004) as implemented in MEGA v6.06 (Tamura, Stecher, Peterson, Filipski, & Kumar, 2013). The nucleotide sequence compositions and nonsynonymous (*n*) and synonymous (*s*) substitution rate (dN/dS) were calculated in MEGA v6.06.

The molecular diversity indices for the number of haplotypes (*H*), haplotype diversity (*h*), and nucleotide diversity (*π*) were calculated in DnaSP v5.10.1 (Librado & Rozas, 2009). A substitution model for the haplotype dataset was determined using the Akaike Information Criterion (AIC) in jModeltest v2.2.10 (Posada, 2008). The TrN model of evolution with the gamma shape parameter and proportion of invariable sites (TrN + I + G) was selected for the analysis of molecular variances (AMOVA) and phylogenetic analysis.

#### 2.4.2 Phylogenetic and network analyses

Phylogenetic relationships among the mitochondrial ND5 haplotypes were reconstructed using Bayesian inference (BI), maximum likelihood (ML), and maximum parsimony (MP) methods. For the BI and ML analyses, the TrN + I + G model was the most appropriate model of nucleotide substitution. The congeneric species *Gyrodactylus parvae *You, Easy & Cone, 2008 was selected as the outgroup (Ye et al., 2017). MP analysis was implemented in PAUP 4.0b10a (Swofford, 2002). Heuristic searches with tree‐bisection‐reconnection were executed for 1,000 random addition replicates with all characters treated as unordered and equally weighted. ML analysis was conducted using RAxML7.2.8 (Stamatakis, Ludwig, & Meier, 2005), with bootstrap analysis performed with 1,000 replicates. BI analysis was performed using MrBayes3.1.2 (Ronquist & Huelsenbeck, 2003), and one set of four chains was allowed to run simultaneously for 8 million generations. The trees were sampled every 1,000 generations, with the first 25% being discarded as burn‐in. Stationarity was considered to be reached when the average standard deviation of split frequencies was below 0.01. A haplotype network was then constructed using the median‐joining (MJ) network approach with MP Calculation in Network 5.0.1.1 (Bandelt, Forster, & Rohl, 1999).

#### 2.4.3 Population genetic structure

Genetic differentiation among populations was assessed using *F*_st_ pairwise comparisons. The molecular variance was partitioned among groups (*F*_ct_), among populations within groups (*F*_sc_), and within populations (*F*_st_) in Arlequin v3.5.1.2 (Excoffier & Lischer, 2010). The mean genetic distances among lineages were calculated by an uncorrected p‐distance model in MEGA v6.06 (Tamura et al., 2013). In addition, the correlations between the genetic differentiation values of *F*_st_ and geographic distance (straight line) of the sample localities were analyzed to test for isolation by distance (IBD) (Slatkin, 1993). The strength and significance of the relationships between genetic differentiation and geographic distance were assessed using linear regression in Graph Pad Prism v 5.0 (www. graphpad. com).

#### 2.4.4 Divergence time estimation

Divergence time estimation based on the strict molecular clock model was performed in BEAST v1.6.1 (Drummond & Rambaut, 2007). The divergence times of lineages were estimated using the TN93 + I + G model and a Yule speciation tree prior because of the absence of fossil or geological data for calibration. Given that the generation time for gyrodactylids may be as short as 2 days (Bakke et al., 2007), which is considerably shorter than that of other taxa of monogeneans and may result in more rapid divergence at mitochondrial loci. The intermediate mutation rate of 13% per million years ago (Mya) was adopted, as it has been employed for mitochondrial gene analysis in Gyrodactylidae species (Lumme et al., 2016; Pettersen et al., 2015). Analyses were performed for 8 million generations while sampling every 1,000th tree, and the first 10% of trees sampled were treated as burn‐in. The estimates and convergence of effective sample size for all parameters larger than 200 were checked with Tracer 1.7 (Rambaut, Drummond, Xie, Baele, & Suchard, 2018). All resulting trees were then combined with LogCombiner v1.7.3 (Drummond & Rambaut, 2007), with a burn‐in of 25%. A maximum credibility tree was then produced using TreeAnnotator v1.5.3 (Drummond & Rambaut, 2007) and visualized in FigTree v1.4.2 (Rambaut, 2014).

#### 2.4.5 Demographic history

Three methods were used with the ND5 haplotype dataset to trace the demographic history of three lineages. The neutrality test between Tajima's *D* (Tajima, 1989) and Fu's *Fs* (Fu & Li, 1993) was used to test for neutral evolution in Arlequin v3.5.1.2 (Excoffier & Lischer, 2010), with significantly negative values indicating population expansion. The mismatch distribution was calculated in Arlequin v3.5.1.2 to test for signals of demographic expansion with a smooth unimodal curve (Harpending, 1994), and the significance of the sum of squared deviations (SSD) and Harpending's raggedness index (Hri) evaluated out by bootstrap resampling (10,000 replicates). The beginning time of expansion (t) was calculated in accordance with a previous study (Lumme et al., 2016). Bayesian skyline plot (BSP) analysis was applied in BEAST v1.6.1 (Drummond & Rambaut, 2007) to describe demographic history by assessing the time variation of effective population size. This analysis was performed using the TN93 + I + G substitution model with a mutation rate of 13% per Mya and 8 million generations.

## 3 RESULTS

A total of 79 sequences from eleven geographic populations of *G. konovalovi* was obtained (Figure 2). The sequence alignment provided a dataset matrix of 528 bp, of which 84 bp (15.9%) were parsimony informative sites. Base frequency was biased with the AT content reaching 68.2%. A total of 25 haplotypes were identified among the 79 sequences (Tables 1, 2, 3, 4). The haplotypes exhibited distinct geographical distributions with 18 unique haplotypes and 7 shared haplotypes. The substitution rate (dN/dS) was 0.068, indicating strong purifying selection against nonsynonymous substitutions. All haplotype sequences were deposited in GenBank under accession numbers MN270015–MN270039.

**Table 2:** Mean distance of ND5 haplotypes between lineages of *Gyrodactylus konovalovi*

Lineages	Lineage B	Lineage C	Lineage A
Lineage B			
Lineage C	0.032		
Lineage A	0.083	0.092	

**Table 3:** Results of hierarchical analysis of molecular variance (AMOVA) based on haplotypes of *Gyrodactylus konovalovi*

Source of variation	Degree of freedom	Sum of squares	Variance components	Percentage of variation	Fixation indices
Among groups	2	691.962	15.42611 Va	75.09	*F*_ct_ * = 0.75093
Among populations	8	173.587	2.73934 Vb	13.33	*F*_sc_ * = 0.53539
Within populations	68	161.649	2.37719 Vc	11.57	*F*_st_ * = 0.88428
Total	78	1,027.177	20.54264		

* Significant level *p* < .01.

**Table 4:** Statistics for genetic diversity, neutrality test, mismatch analysis and the time of the expansion based on lineages of ND5 haplotypes of *Gyrodactylus konovalovi*

Lineages	*h*	π	Hri	SSD	Tajima's *D*	Fu's *Fs*	tau	*t* (Mya)
Lineage A	0.791 ± 0.067	0.00939 ± 0.00111	0.16773	0.06752	1.70587	2.68491	9.74219	0.07234
Lineage B	0.669 ± 0.059	0.00292 ± 0.00024	0.20334	0.05733	2.09605	2.14426	3.27148	0.02429
Lineage C	0.934 ± 0.014	0.01728 ± 0.00120	0.03493	0.03333**	0.76689	0.33106	14.78906	0.10981
Total	0.955 ± 0.007	0.04984 ± 0.00458	0.01933	0.03202	1.6579	6.93069	0.00000	‐

Abbreviations: *h*, haplotype diversity; π, nucleotide diversity; Hri, harpending's raggedness index; SSD, sum of squared deviation; tau, expansion parameter; *t*, beginning a time of expansion; Mya, million years ago.

* Significant level *p* < .05.

** Significant level *p* < .01.

**Figure 3:**
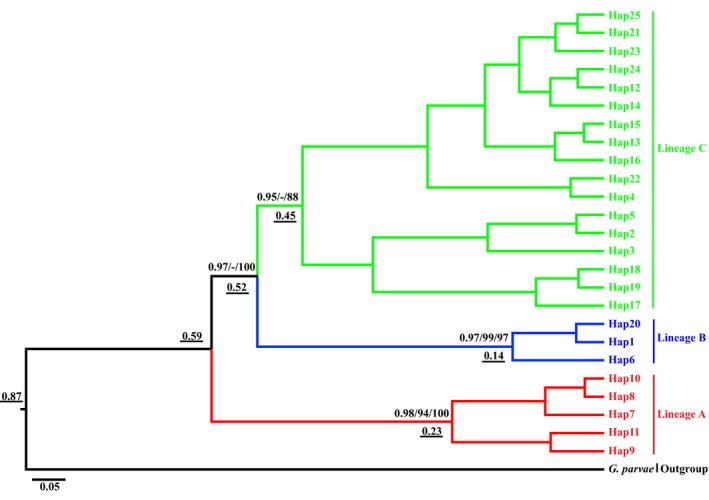
Bayesian inference tree of haplotypes based on the ND5 sequences of *G. konovalovi*. The numbers above nodes are Bayesian posterior probabilities, maximum likelihood (ML) and maximum parsimony (MP) bootstrap values, respectively (those above 50% are shown). The three lineages are differentiated by different colors (red, Lineage A; blue, Lineage B; green, Lineage C). Estimated divergent dates in Mya are given in numbers down nodes with underline

The phylogenetic trees (BI, Ml and MP) based on the haplotype sequences showed congruent topologies and were all divided into three lineages (the BI tree is shown in Figure 3). Lineage A, at the base of the tree was comprised of three populations (HMH, LCH, LYG) from the Han River tributary in the eastern Qinling Mountains, and it exhibited high mean genetic distances that were higher than those of the other lineages (0.083–0.092) (Table 2). Lineage B, corresponding to the Han River tributary, was the sister lineage to lineage C, together distributed in the middle Qinling Mountains, and their genetic distance was 0.032 (Table 2). Lineage C, representing the Wei River and Han River tributaries, included two shared haplotypes (Hap 1 and Hap 7) from GHJ and TTG populations that were corresponded to lineages A and B, showing limited gene flow in adjacent to the vicariance region because of drainage capture. This pattern conformed to obvious geographic distribution and genetic differentiation in the Qinling Mountains.

**Figure 4:**
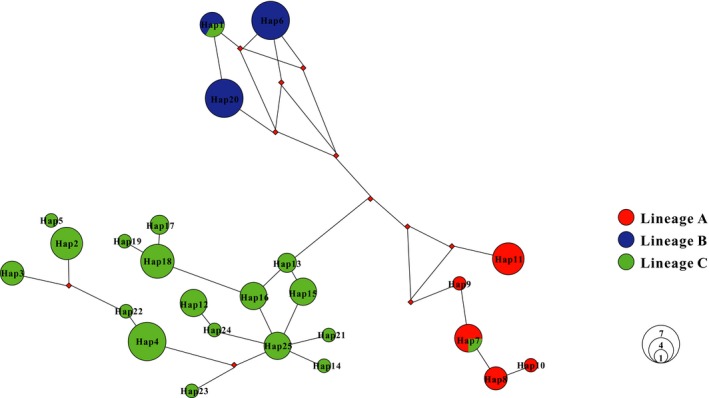
Median‐joining network for all haplotypes of *Gyrodactylus konovalovi* based on the ND5 gene. The colors correspond to lineages as Figure 3

Network analysis revealed that all haplotypes were consistently well‐supported by three different lineages (Figure 4), indicating a significant geographic distribution pattern. All unique haplotypes were connected in a reticulate manner. These relationships revealed a phylogeographic pattern of *G. konovalovi* in the Qinling Mountains in central China.

## 4 DISCUSSION

### 4.1 Genetic diversity

Total haplotype diversity (*h*) and genetic diversity (*π*) across the samples were 0.955 ± 0.007 and 0.04984 ± 0.00458, respectively, indicating high level of genetic diversity. The *h* was highest in the YP population, whereas *π* was highest in the TTG population. The *h* and *π* were lowest in the XYB population, except for three populations (HDT, LCH and LYG), and the relationship between genetic diversity and the population latitude was uncorrelated (Table 1). The populations of *G. konovalovi* showed high levels of genetic diversity, with the mostly unique haplotypes and partially shared haplotypes, indicating limited gene flow adjacent to the vicariance region. Vicariance and uplift promote population genetic differentiation and subdivision and confirming that drainage capture accelerates freshwater fish distribution and dispersal (Albert & Crampton, 2010; Albert, Schoolmaster, Tagliacollo, & Duke‐Sylvester, 2017; Zhang & Chen, 1997; Zhang & Fang, 2012). Dispersal and gene flow between populations of host‐specific fish parasites with a direct life cycle depends on the dispersal of the fish host (Criscione & Blouin, 2004). Barriers to fish upstream migration will reduce the genetic exchange between parasite populations and result in separated populations (Blasco‐Costa, Waters, & Poulin, 2012). The population genetic diversity values with zero are suspected to cause genetic drift by effective population size decline and resulted in bottleneck, founder effect and habitat fragmentation, finally inducing inbreeding, and gene flow deficiency (Hunter & Gibbs, 2006). Vicariance and drainage capture affected population genetic diversity. In this study, the genetic diversity did not show a significant south to north decreasing trend among populations of this parasite in accordance with observations of *F. quadranus* and *R. oxycephalus* in the Qinling Mountains and *G. arcuatus* and *G. gondae* in Europe (Huyse et al., 2017; Lumme et al., 2016; Wang et al., 2012; Yu et al., 2014), indicating the existence of multiple refugia among the Qinling Mountains during the Last Glacial Maximum (LGM). In Europe and North America, species expanded from southern refugia after the LGM (Rowe, Heske, Brown, & Paige, 2004). The genetic diversity of these species is usually geographically structured with declines in genetic diversity toward the north (Hewitt, 1996). The genetic diversity of the marine parasite *Mazocraeoides gonialosae* also displayed a decreasing tendency from south to north along the coasts of the South and East China Seas (Li et al., 2011). However, populations with high haplotype diversity and low nucleotide diversity of *M. gonialosae* and *G. thymalli* and a star‐shaped haplotype network are indicative of a classic postglacial expansion after a period of low effective population size, with rapid population growth enhancing the retention of new mutations, accumulating haplotype diversity, but lacking enough time to accumulate nucleotide diversity (Grant & Bowen, 1998; Hewitt, 1996).

Gene flow was considerably higher among oviparous and viviparous monogenean parasites that were studied along the coasts of the South and East China Seas and the Glomma River in Norway (Li et al., 2011; Pettersen et al., 2015). Such restricted gene flow and most unique haplotypes might cause mutations to persist in isolated populations that have been separated for a long time. A small population might lead to a strong genetic drift, and the reduced genetic diversity due to bottleneck increased genetic drift might increase inbreeding and genetic homogeneity to decrease population fitness (Huyse et al., 2017; Mills, 2007).

Sequence divergence among haplotypes based on the ND5 gene ranging from 0.2% to 12.1% was clearly higher than that found in the monogenean parasites *M. gonialosae* (from 0.01% to 2.08%) and *G. truttae* (0.1% to 0.5%) and approximating *G. teuchis* (0.1% to 11%) and *G. corydori* (0.1% to 6.2%) (Bueno‐Silva et al., 2011; Hahn, Weiss, Stojanovski, & Bachmann, 2015; Li et al., 2011). These values could represent a recent separation.

The substitution rate (dN/dS) was 0.068, indicating strong purifying selection with genes of relatively low nucleotide diversity (*π*) against nonsynonymous substitutions in accordance with results from other gyrodactylids based on COI and ND5 genes, such as *G. thymalli*, *G. salaris*, *G. teuchis,* and *G. arcuatus* (Lumme et al., 2016; Meinilä, Kuusela, Ziętara, & Lumme, 2004; Pettersen et al., 2015). The low values of the substitution rate (dN/dS) noted among all the protein‐coding genes below 0.1 demonstrate that each gene is subject to strong purifying selection, in which the biological functions of proteins are safeguarded against deleterious mutations (Huyse, Buchmann, & Littlewood, 2008); this feature is a typical of mitochondrial protein‐coding genes (Rand & Kann, 1998). However, the dN/dS ratio of COI was greater than one (dN/dS > 1), which may indicate that this gene has been under positive selection from *G. corydori* in Brazil (Bueno‐Silva et al., 2011).

### 4.2 Phylogenetic and network analyses

The topologies of the phylogenetic trees and network analyses yielded similar results, both indicating well‐supported different lineages, revealing obvious phylogeographical pattern in the Qinling Mountains. The pattern clearly conformed to the geographic structure of the Qinling Mountains. The Qinling Mountains have played important roles in influencing the phylogeography of amphibian and fish species and forming obvious geographic structures (Bowen et al., 2016; Liu et al., 2015; Lumme et al., 2016; Meng, Li, & Qiao, 2014; Wang et al., 2012, 2013; Yu et al., 2014), suggesting a historical vicariance pattern.

### 4.3 Population genetic structure

**Figure 5:**
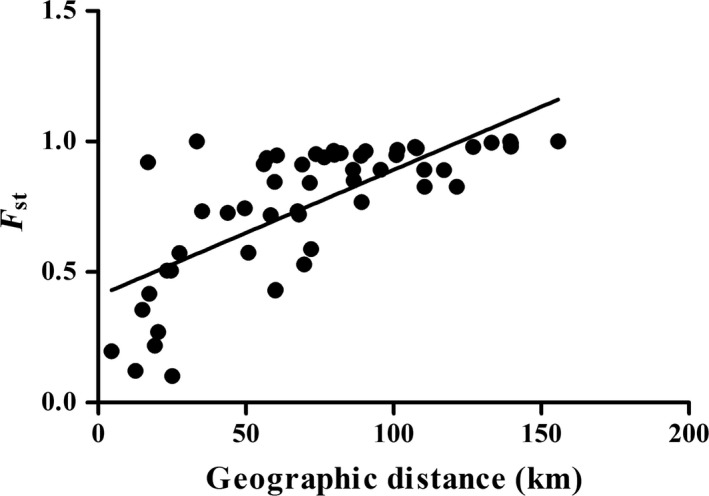
Plots of genetic differentiation estimates of *F*_st_ against geographic distance (km) between populations within the ND5 dataset of *Gyrodactylus konovalovi*. The linear regression overlays the scatter plots (*r* = .7222, *p* < .001)

Analysis of molecular variances (AMOVA) indicated highly significant genetic differentiation among groups assigned to three lineages (75.09%), among populations (13.33%) and within populations (11.57%). The genetic differentiation metric *F*_st_ (0.88428) was very high within populations (Table 3). Furthermore, there was a significant positive correlation between genetic differentiation (*F*_st_) and geographic distance (*r* = 0.7222, *p* < .001) (Figure 5). The AMOVA indicated strong geographic structuring with significant genetic differentiation among populations. This pattern conformed to obviously geographic structure and genetic differentiation in the Qinling Mountains. The Qinling Mountains have played important roles in influencing the phylogeography of amphibian and fish species and forming obvious geographic structures (Bowen et al., 2016; Liu et al., 2015; Lumme et al., 2016; Meng et al., 2014; Wang et al., 2012, 2013; Yu et al., 2014), suggesting a historical vicariance pattern. The orogeny of the Qinling Mountains may have initiated the divergence into independent lineages (Yan, Wang, Chang, Ji, & Zhou, 2010). Thus, barriers to gene flow produced by complex geological history appear to be responsible for driving the high level of species diversity in these mountains in China. These mountains represent a natural boundary between the north and the south, dividing the Chinese temperate and subtropical climatic zones (Ding et al., 2013) and resulting in differentiated terrestrial and freshwater fauna (Li, 1981; Zhang, 2011). The host *R. lagowskii* and other gyrodactylids also showed high genetic differentiation and strong geographical structure in East Asia, Europe and Brazil (Bueno‐Silva et al., 2011; Hahn et al., 2015; Hassan et al., 2015; Huyse et al., 2017; Lumme et al., 2016; Min & Yang, 1986; Pettersen et al., 2015; Xue et al., 2017). The *M. gonialosae* results showed a high rate of gene flow between different populations and a lack of genetic structure because of the continuous distribution in marine environments (Li et al., 2011). Furthermore, there was a significant positive correlation between genetic differentiation (*F*_st_) and geographic distance fit the IBD model in accordance with other studies (Meng et al., 2014; Pettersen et al., 2015; Yu et al., 2014), suggesting that the distribution of genetic variation is due to geographical separation, rather than natural selection. The population genetic differentiation within *F. taihangnica* and *Odorrana schmackeri* rejected the IBD model (Li et al., 2011, 2015; Wang et al., 2013), indicating high gene flow and panmixia in these species. The remarkable spatial genetic differentiations among *F. taihangnica* lineages likely reflect the effects of historical isolations induced by tectonic changes and Pleistocene climatic changes (Wang et al., 2013). More distant sites within the hydrological network were more genetically differentiated in the mountains. The host of this ectoparasite primarily occurs in the clear cold freshwater from middle steam to upstream (Kang, Min, & Yang, 2000; Nishida et al., 2015). This specific ecological habitat results in small population sizes and restricts gene flow among populations (with each population possessing unique haplotypes). Therefore, these populations most likely survive in isolated habitats and differentiate through genetic drift and selection.

### 4.4 Divergence time estimation

A strict molecular clock was estimated for the haplotypes (Figure 3). The time since the most recent common ancestor of the whole ingroup was dated to 0.87 Mya. Lineage A diverged 0.59 Mya, and lineages B and C diverged 0.52 Mya as estimated using a mutation rate of 13% per Mya. All divergence times occurred during the middle Pleistocene. The divergence time of the populations during the middle Pleistocene are in accordance with those for the gyrodactylids *G. corydori*, *G. teuchis*, *G. arcuatus,* and *G. gondae* (Bueno‐Silva et al., 2011; Hahn et al., 2015; Huyse et al., 2017; Lumme et al., 2016). In contrast, the divergence time of *G. thymall* from the Glomma River in Norway was estimated as very recent several thousand years during the Holocene after the LGM (Pettersen et al., 2015). The orogeny of the Qinling Mountains may have initiated divergence into the independent lineages (Yan et al., 2010), while the estimated divergence times in the early to middle Pleistocene might correspond to the rapid uplift (by more than 500–1,500 m) of these mountains that was influenced by the Qinghai‐Tibet Plateau movement (Zhang & Fang, 2012). The phylogeographic distribution of freshwater fishes was affected by climate fluctuation during the Pleistocene (Gao et al., 2012), and the phylogeographic distribution of *R. oxycephalus* was affected by geological events and Pliocene climate fluctuations (Yu et al., 2014).

### 4.5 Demographic history

**Figure 6:**
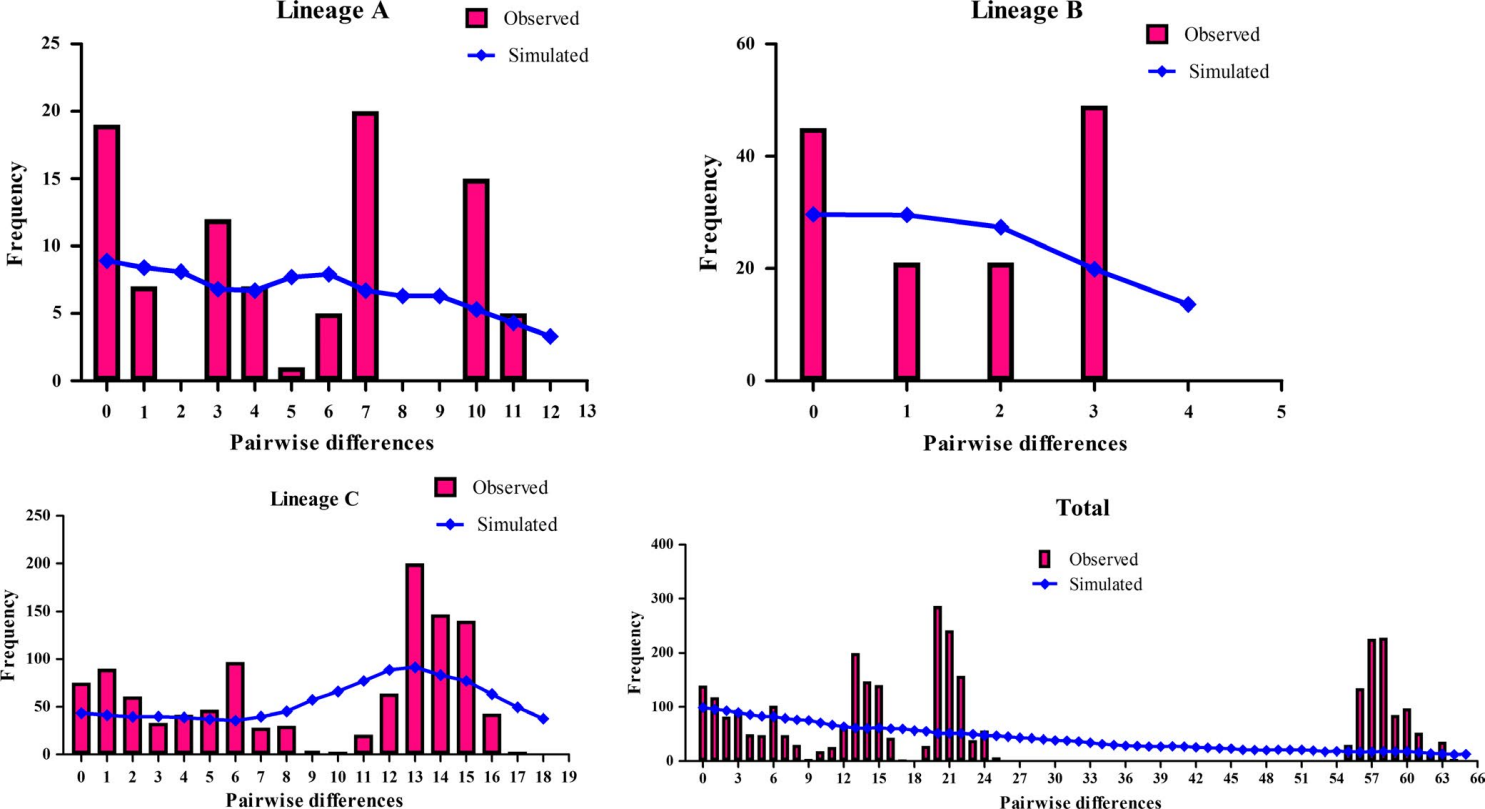
Mismatch distributions for three lineages and the total population of *Gyrodactylus konovalovi*. The observed pairwise differences are shown as red bars, and the simulated values under the sudden expansion model are indicated by blue solid lines

**Figure 7:**
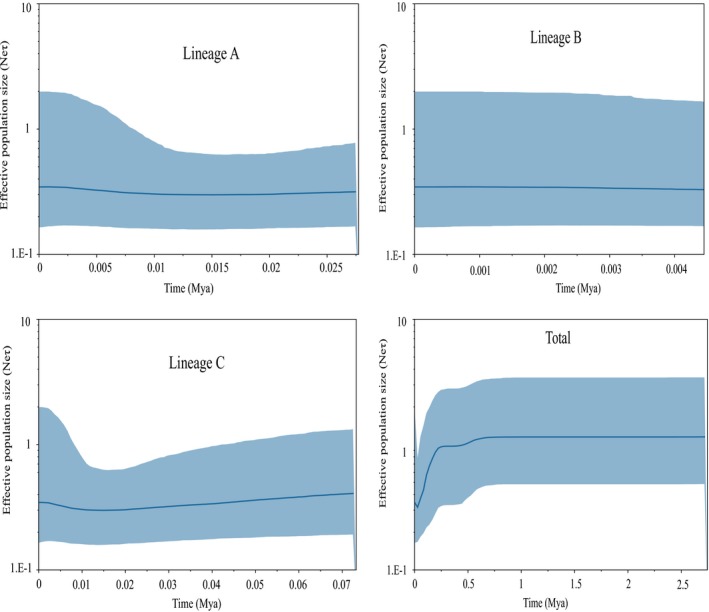
Bayesian skyline plot estimated for the demographic patterns of each lineage and the total population of *Gyrodactylus konovalovi*. The X‐axis represents time in millions of years, and the Y‐axis represents effective population size. The solid line represents the median value of population size, and the dashed lines represent the 95% higher posterior density

The neutrality test between Tajima's *D* and Fu's *Fs* values was positive and not significant (Table 4). However, the values of the SSD and Hri index of all lineages except lineage C and of the total population did not reject the hypothesis that sudden expansion and mismatch distribution were multimodal (Figure 6). The neutrality test and mismatch distribution, rejected the hypothesis of population expansion, indicating that the populations were stable and did not undergo expansion. The values from the neutrality test for the lineages were both positive and nonsignificant, and the mismatch distribution was multimodal, suggesting a bottleneck effect. The BSP suggested that the effective population size increased slowly for lineages A and C and the total population after approximately 0.01 Mya during the Holocene; however, the total population underwent a steep decline after 0.25 Mya, corresponding to the Pleistocene glacial epoch (Figure 7). Therefore, all lineages and total population were discordant in the demographic scenarios based on three methods. The results of the mismatch distribution, neutrality test and BSP showed that the total population underwent contraction during the end of the middle Pleistocene glacial epoch and with interglacial population expansion during the Holocene. The phylogeography and historical demography of amphibian species were profoundly influenced by climate fluctuations during the Pleistocene and tectonic changes during the late Miocene to late Pleistocene in the Qinling Mountains (Meng et al., 2014; Wang et al., 2013).

Most of demographic analyses indicated that the expansion of lineage A and lineage C began simultaneously, 0.01 Mya after the LMG during the late Pleistocene, which corresponds to the end of Dali glaciation in the Qinling Mountains in accordance with findings in *M. gonialosae*, *G. arcuatus*, *G. gondae,* and *G. corydori* (Bueno‐Silva et al., 2011; Huyse et al., 2017; Li et al., 2011; Lumme et al., 2016). The BSP showed that the total population of *G. konovalovi* underwent a sharp contraction from 0.25 Mya to 0.01 Mya during the late Pleistocene glacial epoch, which corresponds to the end of Lushan glaciation in accordance with *F. taihangnica* in the Qinling Mountains (Huang, 1982; Wang et al., 2013). The values from the neutrality test for the lineages were positive and nonsignificant, and the mismatch distribution was multimodal, suggesting a bottleneck effect which is a sharp reduction in the size due to climate change after the glacial epoch (Lande, 1988). A slightly different form of bottlenecks can occur if a small group becomes geographically separated from the main population, such as through a founder events; such populations are often small and show increased sensitivity to genetic drift, an increased inbreeding, and relatively low genetic variation (Lynch, Conery, & Burger, 1995). Ancient population bottlenecks or founder effects may have reduced genetic diversity. Therefore, the climate fluctuation and tectonic changes that led to vicariance and drainage capture during the Pleistocene affected by the phylogeographic distribution of *G. konovalovi* in the Qinling Mountains.

## 5 CONCLUSIONS

The results of this study showed that the climate fluctuation and tectonic changes led to formed vicariance and drainage capture during the Pleistocene, which promoted lineages divergence and influenced the phylogeographic structure of *G. konovalovi* in the Qinling Mountains. After the LGM, most lineages have experienced expansions of population size and range. The findings provide evidence that Pleistocene climatic changes profoundly affect this species distribution pattern and demographic history in the Qinling Mountains. Further studies employing extensive sampling across larger ranges and multiple molecular markers might provide more insight into the phylogeography and demographic history of *G. konovalovi* across the Qinling Mountains and test coevolutionary relationships between *G. konovalovi* and its host *R. lagowskii* at the population level and the magnifying glass hypothesis.

## CONFLICT OF INTEREST

None Declared.

## AUTHOR CONTRIBUTIONS

You P designed the research; Chen T, Chen J, Tang L, Chen X, Yan J, and You P performed the research; Chen T analyzed the data; Chen T and You P wrote the paper.

## Data Availability

DNA sequences: The haplotype sequences were deposited in GenBank under accession numbers MN270015‐MN270039. The data have been uploaded upload into Dryad under the following https://doi.org/10.5061/dryad.6djh9w0wz.
